# Substantial Differences in the Subgingival Microbiome Measured by 16S Metagenomics According to Periodontitis Status in Older Women

**DOI:** 10.3390/dj6040058

**Published:** 2018-10-19

**Authors:** Michael J. LaMonte, Robert J. Genco, Wei Zheng, Daniel I. McSkimming, Christopher A. Andrews, Kathleen M. Hovey, Lu Li, Yijun Sun, Michael J. Buck, Amy E. Millen, Karen L. Falkner, Jean Wactawski-Wende

**Affiliations:** 1 Department of Epidemiology and Environmental Health, University at Buffalo, 270 Farber Hall, 3435 Main Street, Buffalo, NY 14214, USA; koreilly@buffalo.edu (K.M.H.); aemillen@buffalo.edu (A.E.M.); jww@buffalo.edu (J.W.-W.); 2 Departments of Oral Biology, and Microbiology and Immunology, and Center for Microbiome Research, University at Buffalo, 135 Foster Hall, 3435 Main Street, Buffalo, NY 14214, USA; rjgenco@buffalo.edu; 3 Department of Computer Science and Engineering, University at Buffalo, 338 Davis Hall, Buffalo, NY 14214, USA; Wzheng4@buffalo.edu (W.Z.); lli59@buffalo.edu (L.L.); 4 Genome, Environment, and Microbiome Community of Excellent, University at Buffalo, 3435 Main Street, Buffalo, NY 14214, USA; dim@buffalo.edu; 5 Department of Ophthalmology, University of Michigan, 500 S. State Street, Ann Arbor, MI 48109, USA; candrews@buffalo.edu; 6 Departments of Immunology, Computer Science and Engineering, and Bioinformatics, University at Buffalo, 338 Davis Hall, Buffalo, NY 14214, USA; yijunsun@buffalo.edu; 7 Departments of Biochemistry and Bioinformatics, New York State Center of Excellence in Bioinformatics and Life Sciences, University at Buffalo, 955 Main Street, Suite 4102, Buffalo, NY 14214, USA; mjbuck@buffalo.edu; 8 Department of Oral Biology, University at Buffalo, 135 Foster Hall, 3435 Main Street, Buffalo, NY 14214, USA; falkner@buffalo.edu

**Keywords:** microbiome, periodontal disease, women, menopause, aging

## Abstract

Aging invokes physiological changes, such as immunosenescence and inflammation, that could increase host susceptibility to oral microbiome shifts that enable periodontitis progression in later life. At present, there is a dearth of studies specifically evaluating the oral microbiome and periodontitis in older adults. We used high-throughput untargeted sequencing methods and functional metagenomic analyses to assess and compare the subgingival biofilm of postmenopausal women (mean age 71 years) according to periodontitis status. Subgingival plaque samples were obtained from 15 postmenopausal women with no periodontitis, and from 15 women with severe periodontitis, determined by probing measures. The 16S rRNA gene (V1–V3 region) was sequenced on the 454 FLX platform. The PICRUSt technique was used to provide information on what the potential functional characteristics of microbiota might be in healthy, compared with diseased, periodontium. The subgingival microbiome associated with periodontitis showed clear differences to that associated with health. Of the 464 species identified, 22.8% had elevated abundance in disease, while only 6.3% had elevated abundance in health. Among the 12 most prevalent organisms in periodontitis, one-half have previously been recognized as periodontal pathogens by other investigators. The subgingival microbiome in periodontitis contained genes that could code for specific activities, including microbial mobility, synthesis of endotoxin, and proteolytic degradation. The healthy microbiome included genes that could code for sustaining microbial life, including encoding for transporters, glycolysis, gluconeogenesis, the Krebs cycle, and protein kinases. In the present study on postmenopausal women, aged 60 and older, the subgingival microbiome differed in composition and potential function between those with and without periodontitis. Studies of functional gene expression, such as transcriptomics, are needed to definitively identify the molecules carrying out functions associated with pathogenic subgingival complexes. This, in turn, could lead to identification of targets for enhanced management of periodontitis and, possibly, other diseases, in later life.

## 1. Introduction

Aging is a complex process characterized by progressively lower resilience to stress, and greater susceptibility to pathologic insult and disease onset [1]. Alterations in the host environment that occur with the physiological aging processes could enable untoward shifts in relative abundance of commensal and pathogenic bacteria, and enhanced expression of pathogen genomes which, in turn, could heighten disease susceptibility. The subgingival biofilm harbors a polymicrobial ecology that gives rise to periodontitis in susceptible hosts [2]. In a recent comprehensive review of published literature on the oral microbiome, Feres et al. [3] concluded that the majority of studies have included younger and middle-aged adults, and that only a small number of studies, using low throughput microbial measurement techniques, have described the subgingival microbiome in older adults. No study has compared subgingival microbial composition and diversity in older adults, with and without periodontitis. In this study, we used high-throughput sequencing methods to characterize the subgingival microbiome composition, and used metagenomic analysis to elucidate its potential functional characteristics in health and periodontal disease among postmenopausal women, aged 60 and older.

## 2. Results

### 2.1. Characteristics of Study Group

Participants are described in Table 1. Overall, women in the present study were, on average, 70.5 years of age, all were Caucasian, the majority had at least some college education, and none reported current smoking. Women with periodontitis were older, had lower BMI, were less likely to have postgraduate education, and less likely to report current hormone therapy use compared to women without periodontitis. The mean number of teeth present was 22.1 and 25.7 in women with and without periodontitis, respectively. Whole-mouth mean pocket depth (PD), clinical attachment level (CAL), and percentage of sites bleeding, were each higher in women with, than without periodontitis, as expected.

### 2.2. Microbial Community Structure and Composition

**Figure 1:**
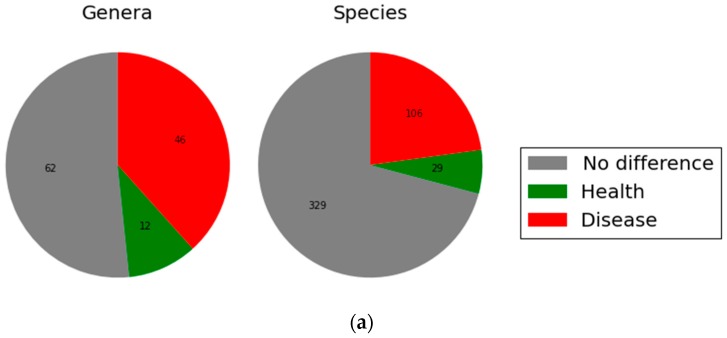

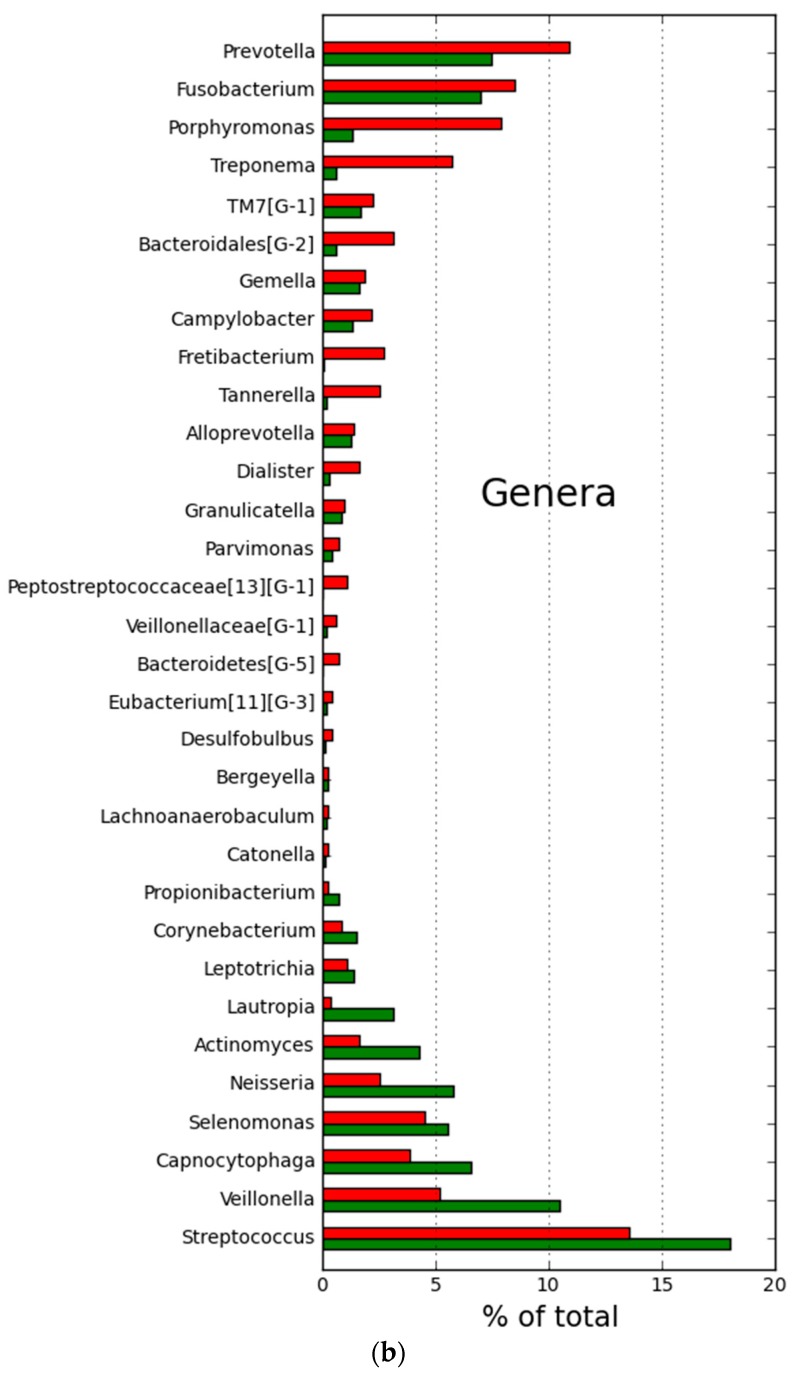

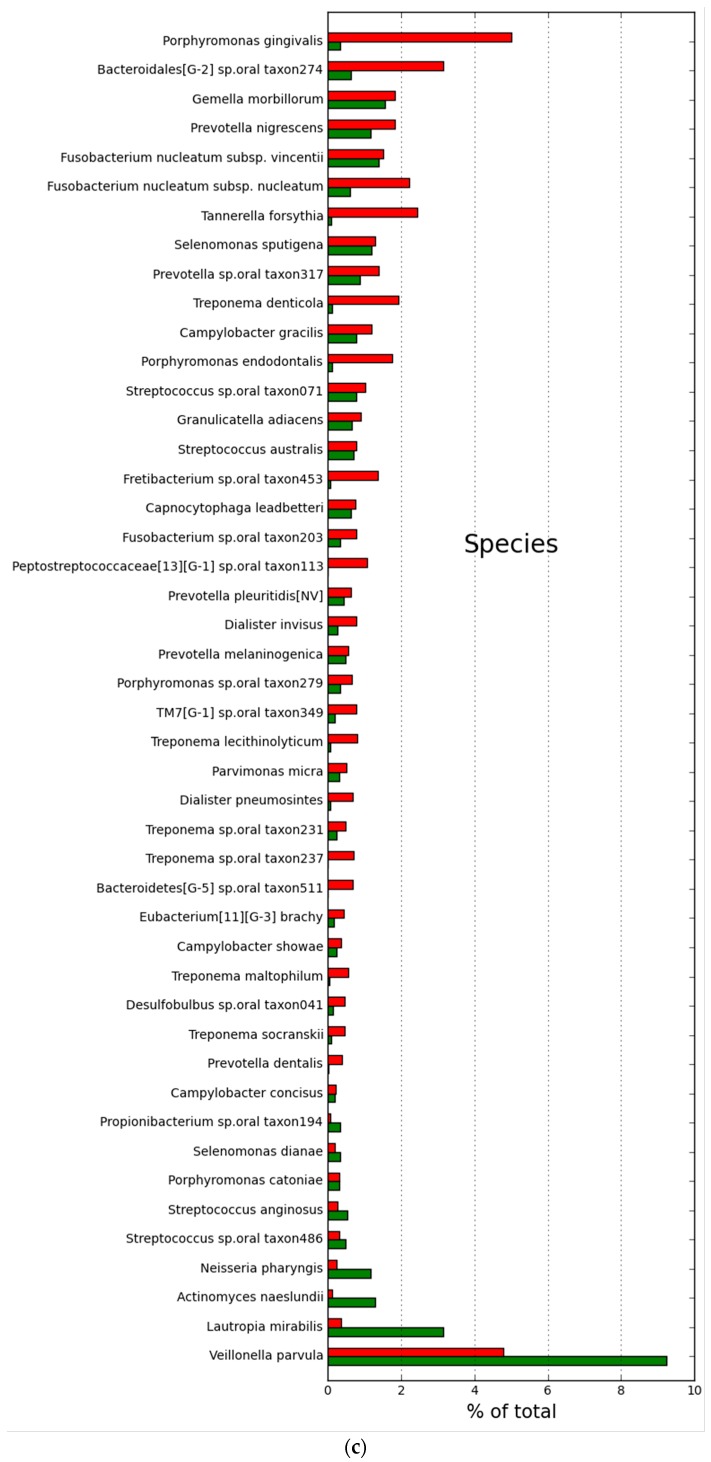
Genus and species level microbial differences between women with and without periodontal disease. (**a**) Panel A. Green = species/genus had significantly higher relative abundance in health; Red = species/genus had significantly higher relative abundance in disease. Disease (red) and health (green) refer to women with and without periodontal disease, respectively; (**b**) Panel B. Genera with an overall relative abundance ≥0.2%, and an adjusted *p* ≤ 0.05, ordered by the difference of relative abundance. Red and green bars are for women with and without periodontal disease, respectively; (**c**) Panel C. Species with an overall relative abundance ≥0.2% and an adjusted *p* ≤ 0.05, ordered by the difference of relative abundance. Red and green bars are for women with and without periodontal disease, respectively.

A general overview of findings at the levels of genus and species, in women with and without periodontitis, is provided in Figure 1. The pie chart (Figure 1. Panel A) shows that, of the 120 genera identified, 46 (38%) had significantly higher relative abundance in disease, whereas 12 (10%) had significantly higher relative abundance in health (adjusted *p* < 0.05). Of the 464 species identified, 106 (22.8%) had significantly higher relative abundance in disease, and 29 (6.3%) had significantly higher relative abundance in health (adjusted *p* < 0.05). The specific genera and species with relative abundance ≥0.2%, and adjusted *p* < 0.05, for comparison between women with and without periodontitis, are shown in Figure 1, Panels B and C, respectively.

**Figure 2:**
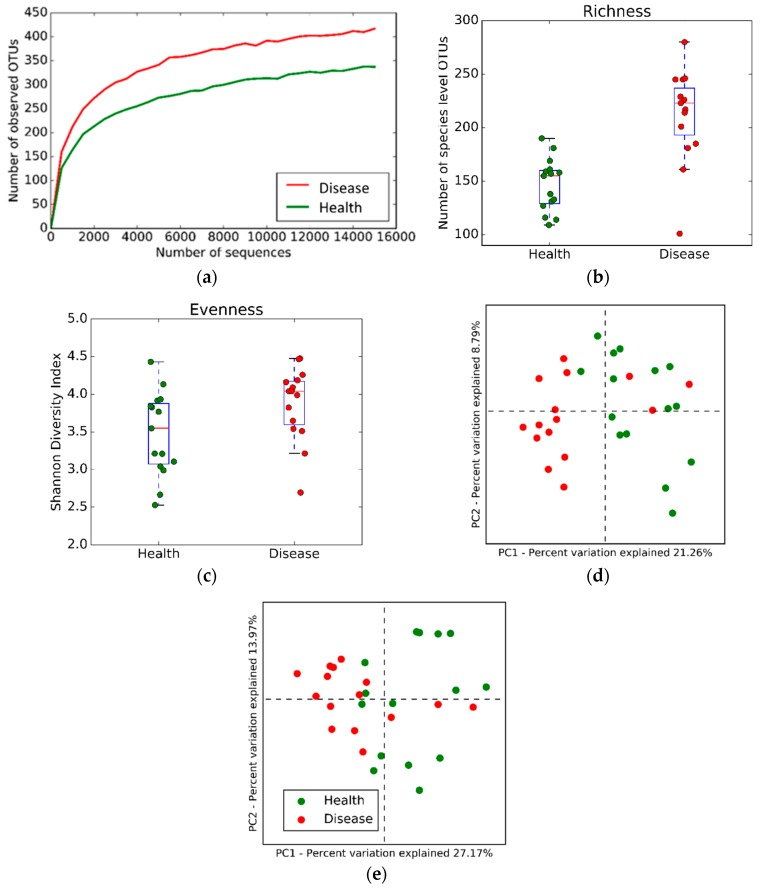
Alpha-diversity and beta-diversity analyses. Alpha-diversity analysis shown in Panels a–c; Beta-diversity in Panels D and E. The rarefaction curve (Panel A) shows a greater number of operational taxonomic units (OTUs) are observed in women with, compared to without periodontal disease. Species richness (Panel B) was greater in women with, than without periodontal disease. No difference in species evenness (Panel C) was observed. (**a**) Panel A. Rarefaction curve; (**b**) Panel B. Species richness plot; (**c**) Panel C. Species evenness plot; (**d**) Panel D. Unweighted UniFrac distance; (**e**) Panel E. Weighted UniFrac distance.

Results of the alpha-diversity analysis are shown in Figure 2. The rarefaction curve (Panel A) shows that, across a range of progressively deeper sequencing reads, a significantly greater number of operational taxonomic units (OTUs) are observed in women with, compared to those without periodontitis. Species richness among observed OTUs was significantly greater in women with, than without periodontitis (Panel B), whereas no difference in species evenness was observed (Shannon diversity index, median = 4.2 periodontitis vs 3.6 health). The beta-diversity analysis (Figure 2) indicated significant differences in bacterial species between women with and without periodontitis in both the unweighted (Panel D; *p* = 0.002) and weighted (Panel E; *p* = 0.0001) UniFrac distances.

**Figure 3:**
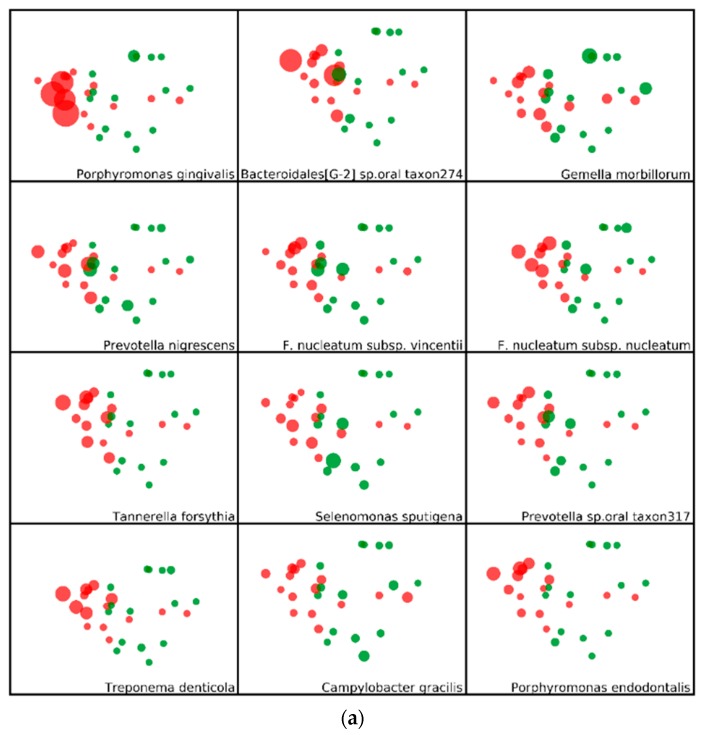

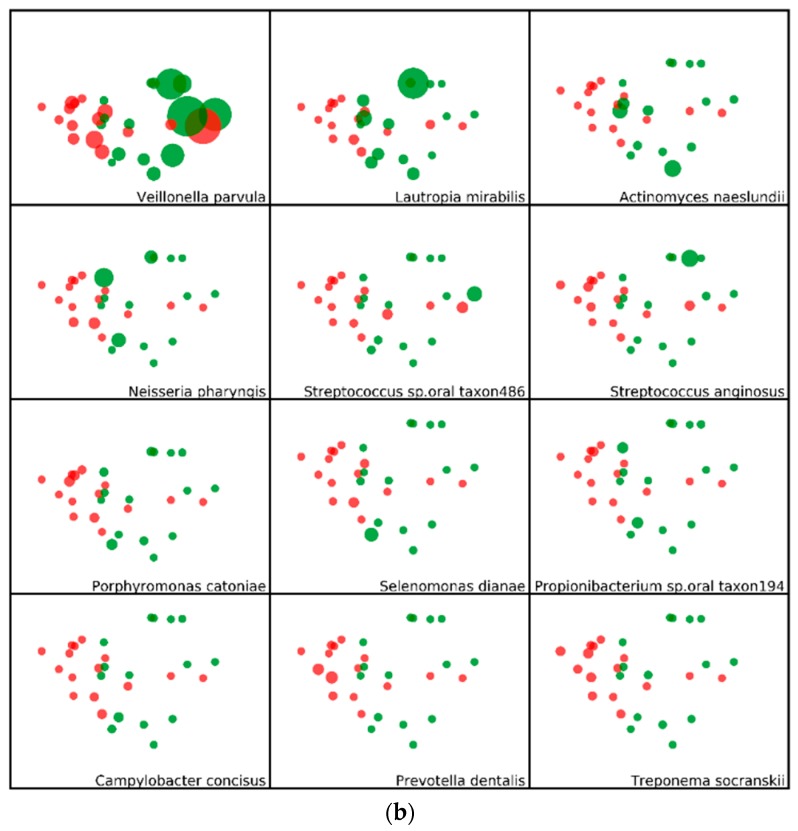
Beta-diversity analysis showing the top 12 bacterial species associated with periodontal disease and with no periodontal disease. The sizes of the datapoints are scaled to reflect the relative abundance of species in each sample. Red and green dots refer to women with and without periodontal disease, respectively. (**a**) Panel A. Bacterial species predominantly associated with periodontal disease; (**b**) Panel B. Bacterial species predominantly associated with no periodontal disease.

To further visualize patterns of subgingival microbiota based on periodontal disease status, we plotted, in Figure 3, the top 12 species in women with (Panel A) and without (Panel B) periodontitis. The size of each datapoint is scaled to their relative abundance within samples. *Porphyromonas gingivalis*, *Bacteroidales*, *Fusobacterium nucleatum*, *Tannerella forsythia*, and *Treponema denticola*, previously described as periodontal pathogens [4], are particularly high in abundance among women with, compared to those without periodontitis. *Veillonella parvula*, *Lautropia mirabillis.*, *Actinomyces naeslundii.*, and *Campylobacter concisus.* are more abundant among women without, compared to those with periodontitis.

### 2.3. Minimum Entropy Decomposition Analysis

**Figure 4:**
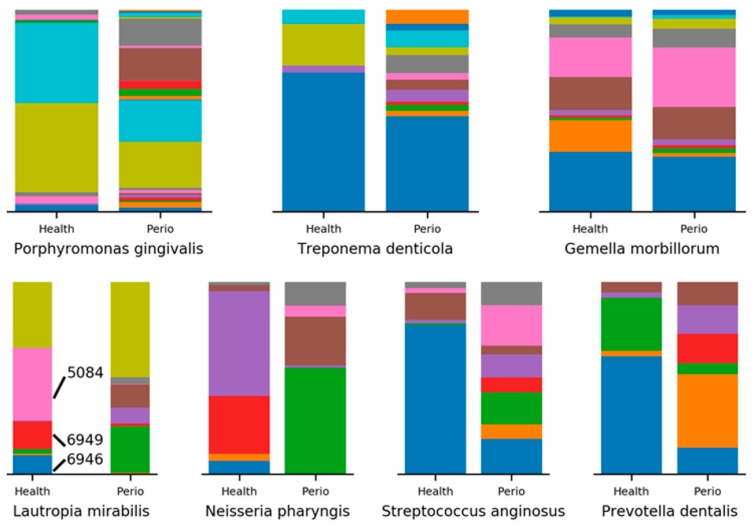
Minimum entropy decomposition (MED) analysis of 9 OTUs with distinct nodes. Examples of subgingival bacteria for which differences in both the number and distribution of MED nodes (e.g., potential subspecies) are seen in periodontal health versus disease.

Next, we sought to increase the resolution with which we viewed the top differentially abundant OTUs through minimum entropy decomposition (MED). Of the top 12 most differentially abundant species in women with periodontitis, 9 had identifiable MED nodes. In general, for each OTU, we see an increase in the number of nodes identified in women with periodontitis, as exhibited by *P. gingivalis* and *T. denticola*. Other OTUs, like *Gemella. morbillorum*, display a more balanced set of MED nodes, with only slight differences in composition between women with and without periodontitis (Figure 4). In the MED analysis of women without periodontitis, we were able to identify 8 of the 12 most differentially abundant species. In contrast to the periodontitis-associated bacteria discussed previously, here, we tend to find more even species richness, with quantitative differences in composition separating women with and without periodontitis. For example, in *L. mirabilis*, 8 of the 9 identified MED nodes are represented in both groups, but three nodes (5084, 6946, 6949) constitute 61.8% of all *L. mirabilis* in women without periodontitis. The same three nodes occupy only 1.6% of *L. mirabilis* in women with periodontitis, a major shift that is accompanied by expansions in four other nodes. Similar trends are observed in *Neisseria pharyngis*, *Streptococcus anginosus*, and *Prevotella dentalis* (Figure 4)*.*

**Figure 5:**
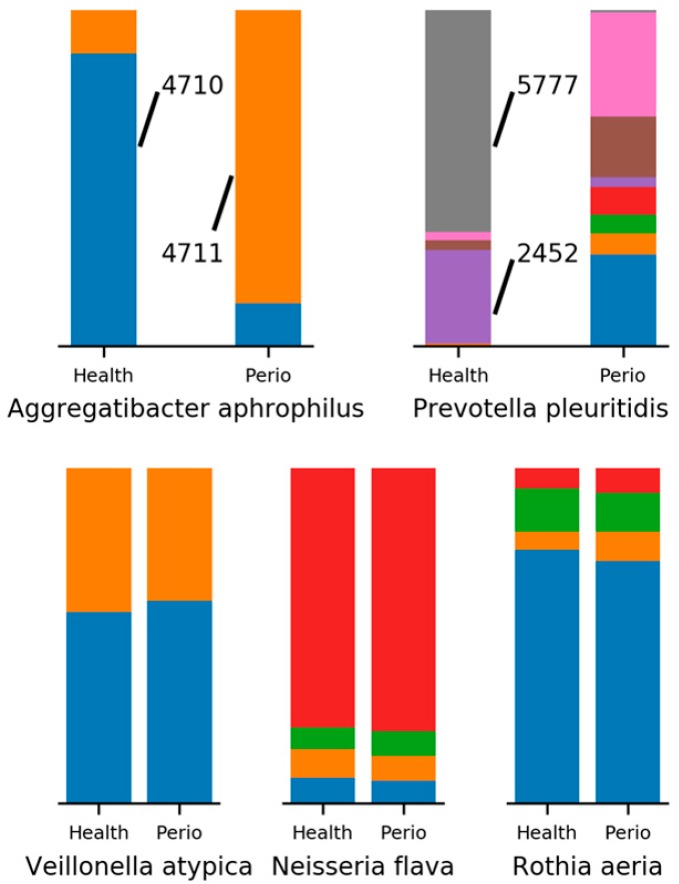
Minimum entropy decomposition (MED) analysis of 5 OTUs with similar nodes. Examples of subgingival bacteria for which there are limited or no differences in the number and distribution of MED nodes (e.g., potential subspecies) in periodontal health versus disease.

Decomposition of OTUs into oligotypes can also reveal interesting differences in OTUs that are not identified as differentially abundant. Perhaps the simplest example occurs in the decomposition of *Aggregatibacter aphrophilus* into its two constituent MED nodes. The first node, 4710, occupies 87.2% of *A. aphrophilus* reads in women without periodontitis, while occupying only 12.7% of reads in women with periodontitis. In contrast, the second node, 4711, shows the exact opposite pattern. *P. pleuritidis* provides a slightly more complicated example, where two nodes (2452 and 5777) occupy 94.1% of reads in healthy women, yet only account for 3.4% of *P. pleuritidis* reads in women with periodontitis (Figure 5). For completeness, we can also examine OTUs such as *Nepenthes flava*, *Veillonella atypica*, and *Rothia aeria*, where both the species richness and composition show little difference between women with and without periodontitis.

### 2.4. Potential Functions of Subgingival Bacteria

**Figure 6:**
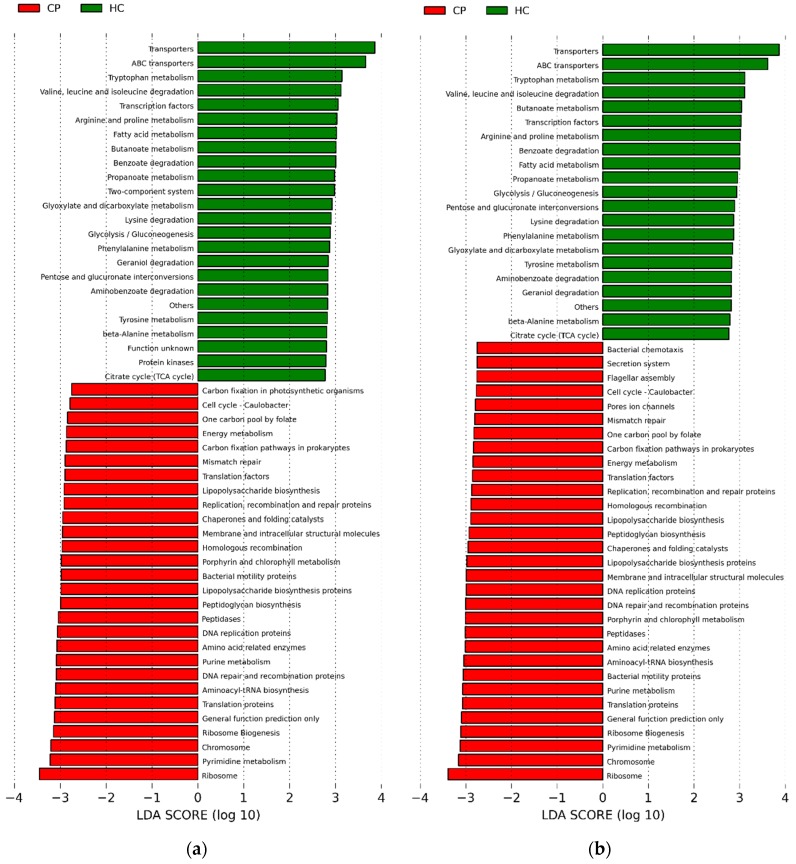
Functional pathways attributed to subgingival biofilm communities. Green bars (HC) are for women with healthy periodontium, Red bars (CP) for women with periodontal disease. (**a**) Panel A. Functional attributes for samples taken from the upper arch; (**b**) Panel B. Functional attributes for samples taken from the lower arch.

Next, we completed an in silico analysis using PICRUSt [4], to speculate on what the functional characteristics of subgingival microbiota might be in health and periodontitis. Linear discriminant analysis (LDA) was used, coupled with effect size measurements (LEfSe) at a cut off of 2.75 (Figure 6). Results showed that there were 34 functional pathways found at statistically higher levels among the genes in subgingival organisms found in periodontitis, as compared to healthy.

## 3. Discussion

Among postmenopausal women, aged 60 and older, the present study showed clear differences in the subgingival microbiome composition in periodontitis compared to health. For bacteria at an abundance of >0.2%, 38% of the genera and 23% of the species identified were at significantly higher relative abundance in women with periodontitis. We also found that 10% of the genera and 6.3% of the species were at higher relative abundance in healthy periodontium. These results are similar to previously reported findings in adults at younger ages [5,6,7,8,9], suggesting there are specific communities of organisms associated with periodontitis, and others with healthy periodontium, that transcend aging. Moreover, several of the specific organisms that were found to be in greater relative abundance in previous studies were also observed in the present study to be at higher relative abundance in older women with periodontitis, suggesting that the current women were infected by similar periodontal pathogenic organisms known from studies on individuals at earlier ages. These organisms include *P. gingivalis*, *F. nucleatum*, *T. forsythia*, and *T. denticola*, each of which are well-documented periodontal pathogens [10]. Other organisms, found in the subgingival microbiota of postmenopausal women in our study, include *G. morbillorum*, *Fretibacterium* taxon 453, *D. invisus* and *pneumosintes*, TM7 (G-1) taxon 349, and *Desulfobulbus* taxon 041. While also reported in studies of younger individuals [3,11], their functional role in periodontitis is unclear, and requires further research using transcriptomics techniques.

The MED analysis, which provides a greater resolution of the microbial composition of a sample by avoiding sequence clustering boundaries, suggests strain level differences may play a role in periodontitis. While well-known oral pathogens, like *P. gingivalis* and *T. denticola*, show increased strain diversity in samples with periodontitis, others, like *L. mirabilis*, *P. dentalis*, and *N. pharyngis*, have drastically altered strain compositions, while maintaining a greater relative abundance in health. Perhaps the most interesting result from the MED analysis involves human pathogens whose relative abundance are not differential between health and periodontitis. Here, we see extreme strain level differences in organisms like *A. aphrophilus* and *P. pleuritidis*, bacteria that have been associated with brain abscess, endocarditis, and pleuritis [12,13,14]. Extreme strain differences between health and disease states suggest functional differences could exist between the strains, possibly acquired through horizontal gene transfer. Future research should identify and sequence the individual strains, and clarify their functional roles in health and periodontal disease.

The α-diversity of the subgingival microbiota in periodontitis differed significantly from that in health. Periodontitis, however, is unlike some other infections where less α-diversity, as well as reduced bacterial species in the diseased state, has been seen. Perhaps a good example of this reduction in complexity is seen in *Clostridium difficile*-associated gastroenterocolitis, where the intestinal bacteria is markedly reduced in complexity, as compared to the bacteria, in the healthy intestine [15]. Pathogenic strains of *C. difficile* represent a major portion of the gut microbiota in diseased individuals. The difference in species richness between a disease like periodontitis and a virulent infection such as *C. difficile* gastroenterocolitis, may reflect a difference in pathogenic mechanisms. In the case of periodontitis, a specific dysbiosis—in which the microbial ecology enriches with motile, anaerobic bacteria capable of generating endotoxins, such as lipopolysaccharide—may be a necessary shift in microbial ecology for development of disease. Several different complexes of organisms may have the same pathogenic functions as part of a multifactorial causal model that leads to periodontitis onset and progression [10,11]. By contrast, in *C. difficile* gastroenterocolitis, the main pathogens appear to be the virulent strains of *C. difficile*, which uniquely produce secreted A and B toxins [15], which appear to be the main cause of this disease.

Using PICRUSt [4] based on whole genome sequences predicted from 16S rRNA sequence information, we conducted an in silico analysis to determine the potential functional roles of the microbiota differing between health and periodontitis. There were over 30 potential functional pathways at statistically elevated levels with an LDA log-score above 2.75 in the genetic pool of subgingival microbiota in periodontitis. Several of the suggested functional pathways are associated with virulence, including endotoxin production, proteolysis, and inflammation. Other investigators have also identified similar functional profiles in subgingival microbiota of adults at younger ages [9,16,17,18,19,20,21].

Kirst et al. [9] published results from a PICRUSt analysis of subgingival 16S sequence data, and they found 13 potential functions significantly elevated at an LDA cut off of 2.75 in periodontitis, findings that are similar to ours. However, we identified an additional 20 potential functions that were overrepresented in the microbiota in periodontitis at the same LDA cut off. The reason for this is unclear. It may be due to a difference in the participants studied. In the present study, postmenopausal women were examined in a controlled clinical setting using calibrated oral probing examination procedures. Kirst and coworkers presumably studied, but did not specify, males and females, who were younger individuals than the women in our study, and they used a different definition of periodontal disease than used herein. Nonetheless, based on speculative results from in silico PICRUSt analysis, there appears to be potential functions suggested in both ours and the Kirst study [9], including bacterial chemotaxis and bacterial mobility proteins, capacity to produce lipopolysaccharide, capacity for protein degradation via production of peptidases, and capacity to express genes related to protein kinases and glycolysis/gluconeogenesis. In periodontitis, genes could potentially code for functions associated with bacterial virulence, such as bacterial chemotaxis, flagellar assembly, lipopolysaccharide biosynthesis, peptidases, and bacterial motility proteins. In healthy periodontium, genes could potentially code for functions associated with maintenance of cellular homeostasis, including ABC transporters, transcription factors, gluconeogenesis, aerobic and anaerobic glycolysis, and protein kinases. In order to definitively characterize the functional differences in microbiota abundant in disease compared with health, and whether the aging process itself influences functional expression by the microbiota, future investigations should use advanced transcriptomics methods to study the oral microbiome and aging.

The strengths of our study are the inclusion of postmenopausal women, an understudied population in dental science, recruited from the community, as opposed to dental or other healthcare clinical settings; the use of untargeted, high-throughput sequencing methods; and, defining health and periodontitis using a standardized whole-mouth oral examination. Limitations include the reliance on in silico analysis of potential functional capacity, using PICRUSt, that differs in microbiota in health and periodontitis. This initial, albeit speculative, approach to understanding function does provide some direction that can be more definitively clarified using advanced transcriptomics and proteomics techniques. The sample size is somewhat small, though comparable with other published studies in this area [8,9,18]. This may have limited achieving statistical significance in a greater number of microbial differences between health and periodontitis. Forthcoming analyses in our overall study cohort will include a larger sample size to further explore and clarify results from this initial analysis.

In summary, we observed taxonomic differences in composition and diversity, and potential functional differences, between subgingival microbiota in health and periodontitis among women in the community, aged 60 and older. Differences in host responses and differences in oral microbial communities have been proposed to account for why periodontitis prevalence and severity may be greater at older ages [3]. However, there has previously been lack of confirmation and description of such differences [3]. The findings reported, herein, begin to address this gap in scientific knowledge. The role that aging has in oral microbiome shifts and, thereby, host susceptibility to periodontitis, is unclear [3], but of substantial relevance to population health, as society undergoes a tremendous increase in adults ages 60 and older [22]. Future studies directly comparing the microbiota composition, diversity, and functional characteristics, between defined samples of older and younger women and men across a range of ages, will be necessary to determine the role that aging has in determining differences in the oral microbiota observed in health and disease. This knowledge could potentially enhance understanding of pathways linking periodontitis with other diseases of aging, such as heart disease, diabetes, and certain cancers [23]. Forthcoming studies in our cohort, which will include longitudinal analysis of changes in subgingival microbiome abundance and diversity in larger numbers of women, will greatly expand understanding beyond which this preliminary report is able to provide.

## 4. Materials and Methods

### 4.1. Participants

The present study included 30 postmenopausal women enrolled in the Buffalo Osteoporosis and Periodontitis (OsteoPerio) Study, which is an ancillary study conducted at the Buffalo (Buffalo, NY, USA) clinical center of the Women’s Health Initiative Observational Study (WHI OS). All protocols for the OsteoPerio Study, and the WHI OS, were approved, annually, by the University at Buffalo Intuitional Review Board. Participants provided written informed consent for all components of the studies, which was conducted in accord with stipulations of the Helsinki Declaration [24]. Details about recruitment, enrollment criteria, and measurements at study entry, have been published for the WHI OS [25] and the OsteoPerio study [26,27]. Briefly, 2249 postmenopausal women, ages 50–79, enrolled into the WHI OS at the Buffalo center in 1993–1998. Of these, 1362 enrolled into the OsteoPerio study in 1997–2001, of whom 1025 completed a follow-up study at Year 5 (2002–2006). Enrollment into the OsteoPerio study required at least 6 teeth present, and no history of bone disease, other than osteoporosis, and no history of cancer in the previous 10 years. Antibiotic use during the previous 90 days and active cancer since baseline were exclusions from the Year 5 visit. 

At both visits, women completed standardized questionnaires pertaining to demographic information, lifestyle habits, personal health history, diet, and medication use, as well as a comprehensive clinical oral examination conducted by trained and calibrated examiners. Based on probing pocket depth (PD) and clinical attachment level (CAL) measures, women were classified on presence and severity of periodontitis using criteria of the Centers for Disease Control and Prevention and the American Academy of Periodontology [28].

For the present study, we randomly selected among women based on their periodontitis status at the Year 5 visit. This study includes 15 women classified as having no periodontitis (<2 interproximal sites with CAL ≥ 4 mm (not on same tooth), and <2 interproximal sites with PD ≥ 5 mm), and 15 women classified as having severe periodontitis (≥2 interproximal sites with CAL ≥ 6 mm (not on same tooth), and ≥1 interproximal site with PD ≥ 5 mm) [28].

### 4.2. Subgingival Plaque Samples

Subgingival plaque samples were obtained at the Year 5 visit by placing fine paper points (#504; Henry Schein, Melville, NY, USA) in the gingival pockets of up to 12 pre-specified teeth (6 maxillary and 6 mandibular teeth) for 10 s. The specific teeth sampled were pre-specified as part of the study’s clinical procedures’ standard of operation, as previously described [29]. Index teeth (3, 5, 7, 9, 12, 14, 19, 21, 23, 25, 28, and 30) were usually sampled. Alternative teeth (2, 4, 8, 10, 13, 15, 18, 20, 24, 26, 29, and 31) were used if the corresponding index tooth was missing. Paper points containing all subgingival plaque samples from each arch were placed directly into 4 mL lactated Ringer’s solution. The solution was then vortexed for dispersion of microorganisms, and then pipetted into 0.5 mL cryovials and frozen immediately at −80 °C, as previously described [29].

### 4.3. 16S rRNA Sequencing, Processing, and Data Analysis

Subgingival plaque samples that had not been thawed after initial collection and freezing were sent by overnight mail on dry ice to The Ohio State University College of Dentistry (Columbus, OH, USA) for 454 pyrosequencing of the subgingival bacterial deoxyribonucleic acid (DNA). The DNA isolation, preparation of the 16S rRNA gene amplicon libraries of the V1–V3 hypervariable regions, PCR amplification, and pyrosequencing using the 454 FLX Sequencer (Roche Diagnostics, Indianapolis, IN, USA) followed the methods described by Griffen et al. [5]. Raw sequence data files were processed using the QIIME pipeline [30] to remove low quality reads. A BLAST search against the Human Oral Microbiome Database (HOMD) version 13.2 [2] was performed. Queries that matched the closest database sequence, at ≥97% identity over an alignment with >90% coverage, were assigned to the respective species-level taxa. We used DESeq2 [31] to determine which taxonomic groups were statistically significantly different between women with and without periodontitis. The Benjamini–Hochberg false discovery rate technique was applied to adjust *p*-values [32]. Microbial diversity between groups was determined using alpha- and beta-diversity analysis [30]. Alpha-diversity analysis consisted of comparisons based on the rarefaction curve, species richness, and evenness. Beta-diversity analysis started with the normalization of the operational taxonomic unit (OTU) table using cumulative sum scaling [30]. Unweighted and weighted UniFrac distances were computed between each pair of samples, and plotted using principal coordinates analysis (PCoA). The ANOSIM test was used to test whether the microbial composition similarity between groups was greater or equal to the similarity within the group. To provide an understanding of the potential functional characteristics of the subgingival microbiota associated with health and periodontitis, we completed an in silico analysis using PICRUSt, a computational algorithm that predicts potential functions based on whole genome sequences derived from 16S rRNA sequence information [4].

## 5. Conclusions

We conclude that, when measured using untargeted high-throughput sequencing technology, the subgingival microbiome differs in composition and diversity between older women with and without periodontitis. Longitudinal studies are needed to further understand the extent to which changes in the subgingival microbiome occur in response to aging, per se, or to other host characteristics of older adults. 

## Figures and Tables

**Table 1:** Participant characteristics for the overall study group and according to periodontal disease status. Data are mean ± SD, or N (%).

Characteristic	Overall	Periodontal Disease Status	
-	-	None	Severe
N	30	15	15
Age (years)	70.5 ± 7.6	67.7 ± 7.3	73.2 ± 7.1
BMI * (kg/m^2^)	25.6 ± 4.8	27.3 ± 5.4	23.9 ± 3.6
Caucasian	30 (100.0)	15 (100.0)	15 (100.0)
Education	-	-	-
High school	8 (26.7)	3 (20.0)	5 (33.3)
College	12 (40.0)	6 (40.0)	4 (26.7)
Postgraduate	10 (33.3)	6 (40.0)	4 (26.7)
Smoking	-	-	-
Never	14 (46.7)	8 (53.3)	6 (40.0)
Former	16 (53.3)	7 (46.7)	9 (60.0)
Current	0	0	0
Hormone therapy use	-	-	-
Never	11 (36.7)	5 (33.3)	6 (40.0)
Former	12 (40.0)	6 (40.0)	6 (40.0)
Current	7 (23.3)	4 (26.7)	3 (20.0)
History of diagnosed treated diabetes	1 (3.3)	0	1 (6.7)
No. teeth present	23.9 ± 3.3	25.7 ± 2.4	22.1 ± 3.1
Whole-mouth PD ** (mm)	2.2 ± 0.6	1.9 ± 0.2	2.5 ± 0.7
Whole-mouth CAL *** (mm)	2.6 ± 0.9	1.8 ± 0.2	3.3 ± 0.8
Sites bleeding on probing (%)	17.0 ± 19.0	9.0 ± 6.0	24.0 ± 25.0

* BMI, body mass index; ** PD, pocket depth; *** CAL, clinical attachment level.

## Availability of Data and Materials:

The datasets used and/or analyzed during the current study are available from the corresponding author on reasonable request.

## Abbreviations

CAL	clinical attachment level
DNA	deoxyribonucleic acid
HOMD	human oral microbiome database
LDA	linear discriminant analysis
MED	minimum entropy decomposition analysis
NGS	next generation sequencing
OS	observational study
OsteoPerio	osteoporosis and periodontal disease study
OTU	operational taxonomic unit
PD	pocket depth
rRNA	ribosomal ribonucleic acid
WHI	women’s health initiative
